# Benefit with preventive noninvasive ventilation in subgroups of patients at high-risk for reintubation: a post hoc analysis

**DOI:** 10.1186/s40560-022-00635-2

**Published:** 2022-09-11

**Authors:** Gonzalo Hernández, Concepción Vaquero, Ramon Ortiz, Laura Colinas, Raul de Pablo, Lourdes Segovia, Maria Luisa Rodriguez, Ana Villasclaras, Juan Francisco Muñoz-Moreno, Fernando Suarez-Sipmann, Alfonso Canabal, Rafael Cuena, Oriol Roca

**Affiliations:** 1 Toledo University Hospital, Toledo, Spain; 2 Ramón y Cajal University Hospital, Madrid, Spain; 3 Ciudad Real General University Hospital, Ciudad Real, Spain; 4 La Princesa University Hospital, Madrid, Spain; 5 Research Unit, Medical Council, Toledo, Spain; 6 Institut de Investigació I Innovació Parc Taulí (I3PT), Parc Tauli Universitari, Sabadell, Spain; 7 Critical Care Department, Alcala de Henares University, Alcala de Henares, Spain; 8 Francisco de Vitoria University, Madrid, Spain; 9 Ciber Enfermedades Respiratorias (CIBERES), Health Institute Carlos III, Madrid, Spain

**Keywords:** Weaning, Postextubation respiratory failure, Reintubation, High-flow conditioned oxygen therapy, Noninvasive ventilation, Outcome

## Abstract

**Background:**

High-flow nasal cannula (HFNC) was shown to be non-inferior to noninvasive ventilation (NIV) for preventing reintubation in a general population of high-risk patients. However, some subgroups of high-risk patients might benefit more from NIV. We aimed to determine whether the presence of many risk factors or overweight (body mass index (BMI) ≥ 25 kg/m^2^) patients could have different response to any preventive therapy, NIV or HFNC in terms of reduced reintubation rate.

**Methods:**

Not pre-specified post hoc analysis of a multicentre, randomized, controlled, non-inferiority trial comparing NFNC and NIV to prevent reintubation in patients at risk for reintubation. The original study included patients with at least 1 risk factor for reintubation.

**Results:**

Among 604 included in the original study, 148 had a BMI ≥ 25 kg/m^2^. When adjusting for potential covariates, patients with ≥ 4 risk factors (208 patients) presented a higher risk for reintubation (OR 3.4 [95%CI 2.16–5.35]). Patients with ≥ 4 risk factors presented lower reintubation rates when treated with preventive NIV (23.9% vs 45.7%; *P* = 0.001). The multivariate analysis of overweight patients, adjusted for covariates, did not present a higher risk for reintubation (OR 1.37 [95%CI 0.82–2.29]). However, those overweight patients presented an increased risk for reintubation when treated with preventive HFNC (OR 2.47 [95%CI 1.18–5.15]).

**Conclusions:**

Patients with ≥ 4 risk factors for reintubation may benefit more from preventive NIV. Based on this result, HFNC may not be the optimal preventive therapy in overweight patients. Specific trials are needed to confirm these results.

**Supplementary Information:**

The online version contains supplementary material available at 10.1186/s40560-022-00635-2.

## Background

Since applying noninvasive ventilation (NIV) as a rescue therapy failed to improve the prognosis of patients with postextubation respiratory failure, management after extubation has focused on preventing reintubation [1]. Supported by evidence from multiple studies [2] and randomized trials [3–9], clinical guidelines recommend that patients with at least one risk factor for reintubation should receive preventive therapy, with either NIV or HFNC immediately after planned extubation [10, 11]. Although superiority over conventional oxygen therapy is widely accepted, the optimal noninvasive supportive therapy to be applied in each patient remains uncertain [12].

It is worth noting that, to date, no model for predicting extubation failure has been validated [13, 14]. Moreover, the definition of high risk for reintubation used in previous studies (≥ 1 risk factor) can result in heterogeneous populations [14, 15]. Some subgroups of high-risk patients benefit with at least partial time on NIV, like those defined by Thille et al. [13, 16, 17] (e.g., aged, obese, patients on mechanical ventilation ≥ 7 days, with ineffective cough or with underlying chronic heart or lung disease) and Ferrer et al. [5] (e.g., patients who develop hypercapnia at the end of the spontaneous breathing trial).

However, the current evidence fails to fully answer two important questions. First, it remains unclear whether some risk factors could benefit more with any specific preventive strategy. Second, while synergistic interactions between risk factors for reintubation have been reported (e.g., advanced age combined with underlying cardiac or respiratory disease, secretions combined with cough strength and neurological status) [18, 19], other possible additive effects of multiple risk factors and how these might affect the response to any preventive therapy remain to be determined [20].

To this end, we performed two post hoc analyses of a non-inferiority randomized clinical trial comparing the preventive effects of HFNC and NIV in patients with high-risk for reintubation [8]. First, we analyzed whether the number of risk factors for reintubation increased the risk of reintubation and benefited more with NIV or HFNC, expecting to find that NIV worked better in patients with greater risk. Second, because the pathophysiological mechanisms involved in HFNC and NIV differ, we analyzed whether the effect on preventing reintubation of HFNC and NIV may be different in obese patients.

Some of the results of this study have been previously reported in the form of an abstract [21].

## Methods

### Study subjects

The design, methods, and population of the trial were fully reported in the original publication [8]. Briefly, the trial tested the hypothesis that HFNC was non-inferior to NIV in by randomizing 604 adult medical and surgical patients in three Spanish intensive care units (ICUs) immediately before planned extubation to receive preventive HFNC or NIV delivered with a total face mask for a fixed period of 24 h and comparing the rates of reintubation and postextubation respiratory failure. Patients were at high risk for extubation failure defined as the presence of at least one of the following: age older than 65 years; heart failure as the primary indication for mechanical ventilation; moderate-to-severe chronic obstructive pulmonary disease; an Acute Physiology and Chronic Health Evaluation II (APACHE II) score higher than 12 on extubation day; body mass index of more than 30; airway patency problems, including high risk of developing laryngeal edema; inability to deal with respiratory secretions (inadequate cough reflex or suctioning > 2 times within 8 h before extubation); difficult or prolonged weaning, in brief, a patient failing the first attempt at disconnection from mechanical ventilation; 2 or more comorbidities according to Charlson score; and mechanical ventilation for more than 7 days. The study protocol was approved by the Departments of Health of the regional governments to which these hospitals are affiliated: Madrid (Comité Ético de Investigación Clínica del Hospital Universitario La Paz, HULP 1/7/10/3116), and Castilla—la Mancha (Comité Ético de Investigación Clínica del Hospital General de Ciudad Real, 28/9/10). All patients or their relatives provided written informed consent and all procedures were followed in accordance with the ethical standards of the regional committees on human experimentation and with the Helsinki Declaration of 1975. The original clinical trial was registered at clinicaltrials.gov (NCT01191489).

The current not pre-planned post hoc subgroup analysis included all 604 patients in the intention-to-treat population. The criteria for reintubation and the criteria to define postextubation respiratory failure are published elsewhere and summarized in the Additional file 1 [8].

### Statistics

Continuous variables were expressed as mean ± standard deviation or median (interquartile range), as appropriate, and qualitative variables as frequency and percentage. Continuous variables were compared using the Student’s *t* test or *U*-Mann–Whitney test, as appropriate. Differences in categorical variables were assessed with the Chi-squared or Fisher’s exact test. Significance was set at 0.05. Statistical analyses were performed using the Stata Statistical Software 14 (StataCorp 15. College Station, TX: StataCorp LP) and SPSS version 13.0 (SPSS Inc.; Chicago, IL).

A multivariate logistic regression model was performed to detect those risk factors with a significant association with the reintubation rate. Sensitivity analysis was performed according to the type of respiratory supportive therapy received after extubation. Forest plot were made using odds ratios and 95% of confidence intervals obtained in the logistic regression analysis.

To assess the effect of the number of high-risk factors on the reintubation rate**,** a univariate analysis was performed including the number of risk factors and taking as a reference the rate of reintubation of those patients with one risk factor. The optimal threshold was decided according to observed the results and finally two separate groups were decided: patients with ≤ 3 and those with ≥ 4 risk factors. To confirm the hypothesis, a multivariate logistic regression analysis was performed including, as a single variable, if the patient has ≥ 4 risk factors and adjusting for other potential covariates (those variables with p < 0.1 when comparing the cohort of patients with ≤ 3 with those with ≥ 4 risk factors, including presence of ≥ 4 risk factors, randomization group, gender, comorbidities, and diagnosis at admission). Additional sensitivity analysis to determine the effect modification for the number of high-risk factors on the reintubation rate for each treatment arm (more detailed explanation in the Additional file 1).

We also analyzed the effect of different noninvasive supportive therapies may differ in the reintubation rate of overweight patients, defined as ≥ 25 kg/m^2^.

Weight in the original study was measured using the integrated system in the ICU beds, while height was measured with a measuring tape while laid on the bed. To assess the effect of overweight on the reintubation rate, a multivariate logistic regression model was performed including body mass index (BMI) ≥ 25 kg/m^2^ and adjusting for all variables with a p value < 0.1 when comparing those patients with BMI ≥ 25 and those with BMI < 25 kg/m^2^ (including BMI ≥ 25, the presence of ≥ 4 risk factors, gender, some risk factors for extubation failure, some comorbidities, and some diagnosis at admission). In addition, as a sensitivity analysis, the same multivariate logistic regression was performed in patients treated with HFNC and in those treated with NIV separately.

## Results

### General characteristics of the population included

Of the 604 patients analyzed, 396 (65%) had ≤ 3 risk factors. The most common number of risk factors was three [*n* = 163 (26.9%)], followed by two [*n* = 158 (26.1%)]. Only 7 (1.1%) patients had 7 risk factors. The most common risk factor was the presence of ≥ 2 comorbidities, 204 (33.7% patients), followed by age, 182 (30.1% patients). Additional file 1: e-Table 1 reports the number of patients with each risk factor according to the number of risk factors the patient had and the treatment the patient received. The total time under NIV was 14 (8–23) hours, whereas the total time with HFNC was 24 (22–24).

### Differences according to the type of respiratory support used after extubation

**Fig. 1:**
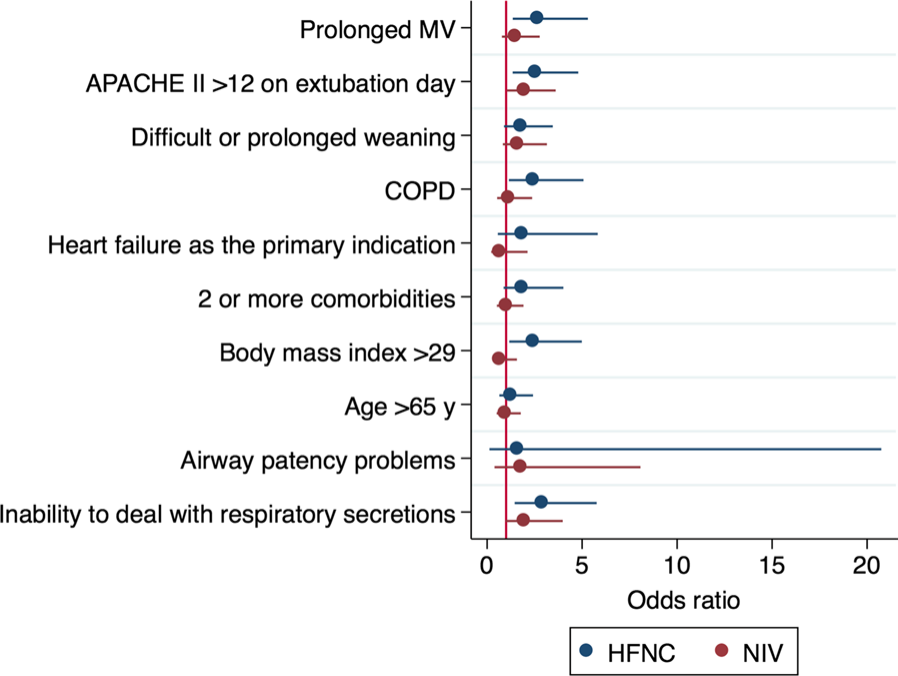
Forest plot of the multivariate logistic regression for reintubation according the type of noninvasive respiratory supportive after extubation (NIV vs HFNC)

The effect of different risk factors on the reintubation rates in the overall population and according to the type of respiratory support used after extubation is presented in the Additional file 1: e-Figure 1 and e-Table 4. Patients with ≥ 4 risk factors presented lower reintubation rates when treated with preventive NIV (23.9% vs 45.7%; *P* = 0.001). Whereas patients with prolonged mechanical ventilation, APACHE > 12 at extubation, COPD, acute heart failure, BMI > 30 and impairment of secretions management had an increased risk for reintubation when they were supported with HFNC, only those with APACHE > 12 presented a higher risk of reintubation when they were treated with NIV (Fig. 1 and Additional file 1: e-Table 5).

## Effect of the number of risk factors on reintubation rate

**Fig. 2:**
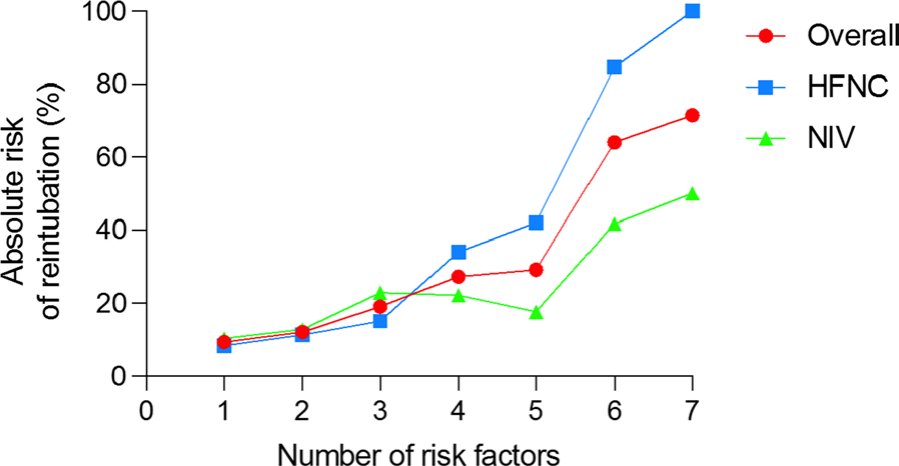
Reintubation rate according to the number of risk factors in the entire population and HFNC and NIV groups separately

Figure 2 shows the reintubation rates according to the number of risk factors. Taking as a reference the rate of reintubation of the patients who had one risk factor, those patients with 4 or more risk factors, presented an increased risk for reintubation even when adjusting for the type of respiratory support used after extubation (see Additional file 1: e-Table 2). The baseline characteristics of the cohort of patients with ≤ 3 vs ≥ 4 risk factors are presented in the Additional file 1: e-Table 3. The presence of ≥ 4 risk factors was independently associated with higher risk of reintubation after adjusting for potential covariates (Table 1). Moreover, similar results were obtained in the sensitivity analysis of the effect modification on reintubation rate because (1) the non-inferiority of HFNC compared to NIV was not confirmed in patients with ≥ 4 risk factors for reintubation, as the one-sided 95%CI calculated for RD did not fulfill the pre-planned criteria [8]; (2) the difference in the RD increased as the number of risk factors increased, with a cutoff of 4 risk factors; and (3) the RD changed from negative to positive and the RR became > 1 in patients with ≥ 4 risk factors (Additional file 1: e-Table 8 and e-Figures 2 and 3).

**Table 1:** Adjusted multivariate logistic regression for reintubation rate according to the presence of ≥ 4 risk factors for reintubation

Variables	OR (95%CI)	*P*
≥ 4 risk factors	3.36 (2.16–5.31)	< 0.001
Randomization	0.72 (.47–1.10)	0.13
Female gender	1.03 (0.66–1.62)	0.88
Comorbidities		
BMI ≥ 25 kg/m^2^	1.11 (0.47–1.10)	0.69
Arterial hypertension	0.65 (0.41–1.04)	0.07
Diabetes mellitus	1.28 (0.79–2.07)	0.32
Vascular disease	2.17 (1.01–4.63)	0.04
Renal disease	0.99 (0.52–1.89)	0.98
Chronic heart disease	0.76 (0.45–1.27)	0.29
Other respiratory disease	1.15 (0.73–1.80)	0.55
Diagnosis at admission		
Pneumonia	2.14 (1.17–3.91)	0.01
COPD exacerbation	0.67 (0.28–1.62)	0.37
Neurologic disease	1.51 (0.88–2.59)	0.13
Cardiologic disease	0.80 (0.40–1.62)	0.54
Trauma	2.24 (0.80–6.26)	0.12
Traumatic brain injury	0.90 (0.25–3.31)	0.88
Surgery	1.00 (0.61–1.66)	0.99

### Effect of overweight on reintubation rate

No differences in reintubation rates between overweight and normal or underweight patients were observed (Additional file 1: e-Table 6). Indeed, after adjusting for potential confounding, overweight was not associated with a higher risk for reintubation (Table 2). However, a sensitivity analysis showed that, patients with overweight had an increased risk for reintubation when they were treated with HFNC (OR 2.47 [95% CI 1.18–5.15]). Oppositely, no increased risk was observed when they were supported with NIV after extubation (OR 0.61 [95% CI 0.27–1.39]).

**Table 2:** Adjusted multivariate logistic regression for reintubation rate in overweight patients

Variables	OR (95%CI)	*P*
BMI ≥ 25	1.37 (0.82–2.29)	0.215
≥ 4 risk factors	1.41 (0.78–2.56)	0.248
APACHE II > 12 on extubation day	1.10 (1.02–1.18)	0.012
Secretions management	2.47 (1.48–4.13)	0.001
≥ 2 comorbidities	1.05 (0.60–1.86)	0.848
Acute heart failure	1.50 (0.52–4.32)	0.449
COPD	1.96 (1.01–3.77)	0.044
Prolonged MV	2.04 (1.25–3.33)	0.004
Female gender	0.90 (0.56–1.44)	0.679
Chronic hepatic disease	1.69 (0.86–3.34)	0.126
Vascular disease	1.54 (0.71–3.30)	0.266
Diabetes mellitus	1.33 (0.81–2.19)	0.258
COPD exacerbation	0.54 (0.20–1.42)	0.215
Trauma	2.01 (0.94–4.29)	0.069
Hemodynamic failure	0.56 (0.25–1.28)	0.176
Neurologic failure	1.43 (0.83–2.44)	0.188

## Discussion

The most important finding of the present study is that traditional definition of high risk for reintubation result in heterogeneous populations, but stratifying this population reveals that outcomes for the two preventive treatments differ according to the number of risk factors and possibly in patients with overweight. Thus, our results reinforce those previously reported in randomized trials supporting the use of NIV over HFNC in some subgroups of patients like those with chronic pulmonary disease, mainly those who develop hypercapnia at the end of the spontaneous breathing trial [5, 16], and patients with chronic heart diseases [16].

Not all risk factors associate the same reintubation rate, making this topic even more complex. Our analysis presented in Additional file 1: e-Figure 1 revealed that prolonged MV, APACHE II at extubation day, not-simple weaning, airway patency problems and secretions management had a stronger association with reintubation. However, a more complex model including these differences could limit its applicability at the bed side.

Considering the number of risk factors for reintubation in analyzing the response to preventive therapies showed that different patients are more likely to benefit from one treatment or the other depending on their level of risk. We found that patients with ≤ 3 risk factors (accounting for 65% of those considered at high-risk under the traditional definition) are likely to have a non-inferior response to preventive HFNC (reintubation rate 12.2%) than to NIV (reintubation rate 16.5%), whereas those with ≥ 4 risk factors are likely to have a better response to preventive NIV (reintubation rate 23.9%) than to HFNC (reintubation rate 45.3%). This result is in accordance to that recently reported by Casey et al. [15] showing an additive effect of simultaneous risk factors and possibly leading to underuse of NIV.

Considering the presence of overweight in analyzing the response to preventive therapies showed that some specific physiological effects of HFNC might be detrimental in this population. The exclusion in the original study of hypercapnic patients reveals that obesity hypoventilation syndrome and the risk for postextubation hypercapnic respiratory failure can be an important mechanism explaining the benefit obtained in a previous study with preventive NIV [17]. Other mechanisms leading to hypoxemia described in overweight patients can be responsible for the worse results observed in our study (e.g., atelectasis after extubation secondary to increased intraabdominal hypertension). Thus, excluding hypercapnic patients our study was underpowered to obtain a better result with NIV. In addition, other differences between the protocol by Thille et al. and ours (e.g., more prolonged preventive therapy according to clinical condition instead of fixed period of 24 h, respectively, different definition of reintubation 7 vs 3 days, respectively, and our higher median number of risk factors), could have led to a better reintubation rate in the HFNC group in the study by Thille et al. (7% vs 33%, respectively). Furthermore, combining HFNC and NIV in the study by Thille et al. led to a 24 h a day prevention protocol while our only NIV led to a 14 h a day in that group, probably limiting efficacy of NIV therapy.

This is a complex topic: obesity has not been associated to an absolute increase in the reintubation rate in previous studies [22], but seems to benefit with preventive NIV [2, 17]. This obesity paradox in weaning could be partially explained with the recent results by Torrini et al. [21] showing that obesity can be a protective factor for reintubation. However, which conditions are necessary to fully obtain that protective effect remains to be elucidated.

### Study limitations

The present study has some limitations. First, it is a post hoc analysis of a non-inferiority trial that can yield false-positive results due to fixed boundaries for pre-planned analyses that include groups with different margins of benefit. The original study defined non-inferiority with a between-group difference in treatment failure < 10%, showing a reintubation rate in NIV patients of 19.1% vs 22.8% in HFNC patients. These findings are currently being tested in a prospective randomized trial (Clinicaltrials.gov ID: NCT04125342). Second, in the absence of a validated model to predict extubation failure, the original study used the ten risk factors for which the most evidence was available. The factors that most increased the reintubation rate in the original trial were prolonged mechanical ventilation, APACHE II score > 12 on the day of extubation, not-simple weaning, and inability to manage respiratory secretions. However, these results are highly dependent on the specific definition of risk factors and are difficult to benchmark. Third, it should be also noted that some specific subgroups of patients were excluded in the original study (e.g., patients who were hypercapnic at the end of the spontaneous breathing trial before extubation). In addition, the retrospective design of this secondary analysis precludes definitive conclusions about the association between any given risk factor and the reintubation rate, as there are no predefined control group for comparison. Fourth, it is likely that the sample size was insufficient to ensure adequate statistical power if all risk factors were included, the adjusted model for the number of risk factors and overweight included only factors associated with reintubation at *P* < 0.10 (APACHE II on extubation day, difficult or prolonged weaning, inability to deal with respiratory secretions, and vascular disease as a comorbidity). Thus, effect modifications and interactions in addition to those related to the number of risk factors and BMI ≥ 25 cannot be ruled out.

## Conclusion

Patients with ≥ 4 risk factors for reintubation could benefit more from preventive NIV. Based on this result, HFNC could not be the optimal preventive therapy in overweight patients. Specific trials are needed to confirm these results.

## Supplementary Information

**Additional file 1.** Supplementary Online Content.

## Data Availability

The datasets used and/or analyzed during the current study are available from the corresponding author on reasonable request.

## Abbreviations

APACHE II: Acute Physiology and Chronic Health Disease Classification System II
BMI: Body mass index
COPD: Chronic obstructive pulmonary disease
HFNC: High-flow nasal cannula
ICU: Intensive care unit
NIV: Noninvasive ventilation
