# Supplementary material for: Benefit with preventive noninvasive ventilation in subgroups of patients at high-risk for reintubation: a post hoc analysis
Source: J Intensive Care. 2022 Sep 11;10:43. doi: 10.1186/s40560-022-00635-2 (PMC9465935; doi:10.1186/s40560-022-00635-2)
Supplement: Supplementary file 1 — Additional file 1. Supplementary Online Content. [file 40560_2022_635_MOESM1_ESM.doc]

**Supplementary Online Content 1**

**Benefit with preventive noninvasive ventilation in subgroups of patients at high-risk for reintubation: a *post hoc* analysis**

Gonzalo Hernández, MD, PhD; Concepción Vaquero, MD; Ramon Ortiz, MD PhD; Laura Colinas, MD; Raul de Pablo, MD PhD; Lourdes Segovia, MD; Maria Luisa Rodriguez, MD; Ana Villasclaras, MD; Juan Francisco Muñoz-Moreno, MD; Fernando Suarez-Sipmann, MD PhD; Alfonso Canabal, MD, PhD; Rafael Cuena, MD; Roca O, MD, PhD.

**This supplementary content includes the following:**

**METHODS**

**1.- Sensitivity analysis:** Effect modification for the number of risk factors on the reintubation rate for each treatment arm.

**2.- Reintubation criteria in the original study.**

**RESULTS:**

**1.- e-Table 1:** Number of patients with each high-risk factor according to the number of risk factors and the therapy applied.

**2.- e-Table 2:** Univariate analysis of the number of risk factors related to the reintubation rate.

**3.- e-Table 3:** Baseline characteristics of intermediate risk (≤3 risk factors) and high risk (≥4 risk factors) groups.

**4.- e-Table 4:** Main outcomes according to stratification of high risk (≤3 risk factors vs ≥4 risk factors).

**5.- e-Table 5:** Multivariate logistic regression model for reintubation including all risk factors in the entire population, the HFNC and NIV groups separately.

**6.- e-Figure 1:** Forest plot of the multivariate logistic regression for reintubation including all risk factors.

**7.- Sensitivity analysis e-Table 6:** Multivariate logistic regression for reintubation including risk factors with significant association with reintubation.

**8.- e-Table 7:** Baseline characteristics of obese (BMI ≥25) and non-obese (BMI <25) patients.

**9.- e-Table 8:** Main outcomes (reintubation and postextubation respiratory failure) according to the stratification of the BMI (≥25 vs <25).

**10.- Sensitivity analysis e-Table 9:** Multivariate logistic regression for reintubation in overweight patients adjusted for covariates.

**11.- Sensitivity analyses:** Effect modification.

**e-Table 10:** Effect modification for the number of risk factors on reintubation rate.

**e-Figure 2.-** Effect modification on an additive scale.

**e-Figure 3.-** Effect modification on a multiplicative scale.

**METHODS**

**1.- Sensitivity analysis:** Effect modification for the number of risk factors on the reintubation rate for each treatment arm.

We calculated relative risks (RR), odds ratios (OR), and risk differences (RD) with two-sided 95% confidence intervals (CI) for each number of risk factors. Moreover, to confirm noninferiority for each category, we also calculated one-sided 95%CIs for RDs. We analysed the effect modification on the additive scale and on the multiplicative scale. We report the effect modification on the additive scale as the relative excess risk due to interaction (RERI) with its respective 95%CI and *p*-value. Values of RERI >0 imply synergism between the exposures. We report the effect modification on the multiplicative scale as the ratio of relative risk (RRR) with its respective 95%CI and *p*-value. Values of RRR >1 imply synergism.

REFERENCES:

1 Knol MJ, VanderWeele TJ. Recommendations for presenting analyses of effect modification and interaction. Int J Epidemiol 2012; 1-7 doi:10.1093/ije/dyr218.

2 Vandenbroucke JP, von Elm E, Altman DG, et al. Strengthening the Reporting of Observational Studies in Epidemiology (STROBE): explanation and elaboration. Epidemiology 2007; **18**: 805–35.

**2.- Reintubation criteria in the original study.**

In this original study the predefined indications for reintubation included:

- Immediate respiratory-related reintubation (any of the following):
  - Respiratory or cardiac arrest.
  - Respiratory pauses with loss of consciousness or gasping for air.
  - Psychomotor agitation inadequately controlled by sedation.
  - Massive aspiration.
  - Persistent inability to remove respiratory secretions.
  - Heart rate less than 50/min with loss of alertness.
  - Severe hemodynamic instability unresponsive to fluids and vasoactive drugs.

In addition, patients who developed persistent postextubation respiratory failure were also reintubated. Persistent postextubation respiratory failure was defined when at least one of the following criteria was fulfilled, after they had undergone the assigned treatment by the treating physician for at least one hour, without fulfilling immediate reintubation criteria:

1. Lack of improvement in pH or in the partial pressure of carbon dioxide or fall in GCS scale >2 points.

2. Lack of improvement in signs suggestive of respiratory-muscle fatigue or worsening including the appearance of unequivocal signs of respiratory-muscle fatigue, such as maintained active contraction of the expiratory muscles, asynchronous motion of the rib cage and abdomen, respiratory alternans, or active contraction of the sternocleidomastoid.

3. Hypotension, with a systolic blood pressure below 90 mm Hg for more than 30 minutes despite adequate volume challenge, use of vasopressors, or both.

4. Copious secretions that could not be adequately cleared or that were associated with acidosis, hypoxemia, and changes in mental status or persistent or worsening signs of respiratory-muscle fatigue.

5. Decrease to SpO2.

**RESULTS:**

**1.- e-Table 1:** Number of patients with each high-risk factor according to the number of risk factors and the therapy applied.

| Risk factor | Number of risk factors (n) | | | | | | |
| --- | --- | --- | --- | --- | --- | --- | --- |
|  | 1 (75) | 2 (158) | 3 (163) | 4 (111) | 5 (65) | 6 (25) | 7  (7) |
|  | NIV/HFNC | | | | | | |
| Prolonged MV | 11/9 | 18/17 | 29/23 | 27/25 | 23/16 | 8/8 | 4/3 |
| APACHE II >12 on extubation day | 1/2 | 15/19 | 28/35 | 41/33 | 28/26 | 12/13 | 4/3 |
| Difficult or prolonged weaning | 3/1 | 6/6 | 20/17 | 25/19 | 19/16 | 10/11 | 4/3 |
| Age >65 y | 2/9 | 41/40 | 51/49 | 51/29 | 23/23 | 10/13 | 4/3 |
| Airway patency problems | 1/3 | 1/1 | 3/1 | 1/0 | 0/0 | 0/1 | 2/0 |
| Inability to deal with respiratory secretions | 2/1 | 4/6 | 19/17 | 18/14 | 14/19 | 6/8 | 3/1 |
| BMI >30 | 2/1 | 11/10 | 18/24 | 12/11 | 11/11 | 6/3 | 2/3 |
| COPD | 2/0 | 5/9 | 20/10 | 21/17 | 11/8 | 6/6 | 1/2 |
| Heart failure as the primary indication for MV | 2/0 | 3/4 | 5/0 | 9/5 | 9/6 | 3/3 | 0/0 |
| ≥2 comorbidities | 13/10 | 50/50 | 57/63 | 50/36 | 32/30 | 11/12 | 4/3 |

APACHE = Acute Physiology and Chronic Health Evaluation; BMI = body mass index; COPD = chronic obstructive pulmonary disease; HFNC = high-flow nasal cannula; MV = mechanical ventilation; NIV = noninvasive ventilation.

The Acute Physiology and Chronic Health Evaluation (APACHE II) was calculated from 17 variables. Scores range from 0 to 71 points, with higher scores indicating more severe disease.

Comorbidities were categorized based on the Charlson Comorbidity Index.

The BMI is weight in kilograms divided by the square of height in meters.

**2.- e-Table 2:** Univariate analysis of the number of risk factors related to the risk of reintubation taking as a reference the reintubation rate of the patients with one risk factor.

| Number of risk factors | Non-adjusted | | Adjusted by therapy | | |
| --- | --- | --- | --- | --- | --- |
|  | OR (95%CI) | P | OR (95%CI) | P |  |
| 2 | 1.32 (.53 – 3.31) | .54 | 1.32 (.53 – 3.29) | .55 | |
| 3 | 2.28 (.95 – 5.44) | .06 | 2.28 (.95 – 5.45) | .06 | |
| 4 | 3.64 (1.50 – 8.81) | .004 | 3.71 (1.53 – 9.00) | .004 | |
| 5 | 4.01 (1.56 – 10.31) | .004 | 4.03 (1.53 – 10.38) | .004 | |
| 6 | 17.26 (5.59 – 53.35) | <.001 | 17.27 (5.58 – 53.44) | .001 | |
| 7 | 24.28 (3.95 – 149.14) | .001 | 24.96 (4.05 – 153.95) | .001 | |

**3.- e-Table 3: Baseline characteristics of intermediate risk (≤3 risk factors) and high risk (≥4 risk factors) groups.**

|  | ≤3 risk factors  (n=395) | ≥4 risk factors  (n=208) | P |
| --- | --- | --- | --- |
| Age, y, median (IQR) | 65 (50 – 75) | 71 (65 – 77) | <.001 |
| Female gender, n (%) | 240 (60.8) | 149 (71.6) | .008 |
| APACHE II at ICU admission, median (IQR) | 15 (12 – 19) | 18 (16 – 22) | <.001 |
| APACHE II the extubation day, median (IQR) | 9 (8 – 12) | 12 (12 – 14) | <.001 |
| Number of high risk factors, median (IQR) | 2 (2 – 3) | 4 (4 – 5) | <.001 |
| Risk factors | | | |
| Prolonged MV (>7 days), n (%) | 107 (27.0) | 114 (55.1) | <.001 |
| APACHE II the extubation day, mean (±SD) | 100 (25.3) | 159 (76.4) | <.001 |
| Not simple weaning, n (%) | 53 (13.4) | 107 (51.7) | <.001 |
| At least moderate COPD, n (%) | 44 (11.1) | 72 (35.0) | <.001 |
| Acute heart failure, n (%) | 13 (3.3) | 34 (16.5) | <.001 |
| Charlson score ≥2, n (%) | 243 (61.4) | 179 (86.1) | <.001 |
| BMI >30, n (%) | 66 (16.7) | 59 (28.6) | .001 |
| >65 y, n (%) | 192 (48.5) | 156 (76.0) | <.001 |
| Airway patency problems, n (%) | 13 (3.3) | 4 (1.9) | .442 |
| Inability to deal with secretions, n (%) | 49 (12.4) | 83 (39.9) | <.001 |
| Type of respiratory support | | | |
| High flow nasal cannula, n (%) | 196 (49.5) | 94 (45.2) | .315 |
| Noninvasive ventilation, n (%) | 200 (50.5) | 114 (54.8) |
| Comorbidites | | | |
| Body mass index >25, n (%) | 79 (20.0) | 69 (33.2) | <.001 |
| Arterial hypertension, n (%) | 205 (51.8) | 136 (65.4) | .001 |
| Chronic heart failure, n (%) | 111 (28.0) | 86 (41.4) | .001 |
| Neurologic disease, n (%) | 104 (26.3) | 53 (25.5) | .835 |
| COPD, n (%) | 49 (12.4) | 75 (36.1) | <.001 |
| Other chronic lung disease, n (%) | 111 (28.0) | 74 (35.6) | .056 |
| Diabetes mellitus, n (%) | 89 (22.5) | 90 (43.3) | <.001 |
| Cancer, n (%) | 71 (17.9) | 42 (20.2) | .498 |
| Chronic vascular disease, n (%) | 18 (4.6) | 25 (12.0) | .001 |
| Chronic renal failure, n (%) | 38 (9.6) | 41 (19.7) | <.001 |
| Chronic hepatic failure, n (%) | 38 (9.6) | 22 (10.6) | .702 |
| Other chronic conditions, n (%) | 52 (13.1) | 29 (13.9) | .781 |
| Reason for mechanical ventilation initiation | | | |
| Respiratory primary failure, n (%) | 124 (31.3) | 95 (45.7) | <.001 |
| ARDS, n (%) | 30 (7.6) | 23 (11.1) | .151 |
| Community adquired pneumonia, n (%) | 44 (11.1) | 41 (19.7) | .004 |
| COPD exacerbation, n (%) | 24 (6.1) | 24 (11.6) | .018 |
| Airway patency roblems, n (%) | 13 (3.3) | 0 (0) | .008 |
| Non respiratory primary failure, n (%) | 252 (63.6) | 157 (75.5) | .003 |
| Neurologic primary failure, n (%) | 109 (27.5) | 34 (16.4) | .002 |
| Heart primary failure, n (%) | 38 (9.6) | 52 (25.0) | <.001 |
| Trauma, n (%) | 44 (11.1) | 8 (3.9) | .002 |
| Trauma brain injury, n (%) | 23 (5.8) | 5 (2.4) | .067 |
| Surgery, n (%) | 165 (41.7) | 67 (32.2) | .023 |
| Non-scheduled surgery, n (%) | 130 (32.8) | 61 (29.3) | .379 |
| Vascular surgery, n (%) | 6 (1.5) | 1 (0.5) | .431 |
| Trauma surgery, n (%) | 6 (1.5) | 1 (0.5) | .431 |
| Cardio-thoracic surgery, n (%) | 5 (1.3) | 0 (0) | .170 |
| Abdominal surgery, n (%) | 67 (16.9) | 42 (20.2) | .320 |
| Facial, neck and ENT surgery, n (%) | 5 (1.3) | 0 (0) | .170 |
| Neurosugical surgery, n (%) | 65 (16.4) | 16 (7.7) | .003 |
| Other, n (%) | 17 (4.3) | 7 (3.4) | .579 |

APACHE = Acute Physiology and Chronic Health Evaluation; ARDS = Acute Respiratory distress syndrome; BMI = body mass index; COPD = chronic obstructive pulmonary disease; HFNC = high-flow nasal cannula; MV = mechanical ventilation; NIV = noninvasive ventilation.

**4.- e-Table 4: Main outcomes (reintubation and postextubation respiratory failure) according to stratification of high risk (≤3 risk factors vs ≥4 risk factors).**

|  | ≤3 risk factors  (n=395) | ≥4 risk factors  (n=208) | P |
| --- | --- | --- | --- |
| All cause reintubation, n (%) | 57 (14.4) | 70 (33.8) | <.001 |
| HFNC group, n (%) | 24 (12.2) | 43 (45.7) |
| NIV group, n (%) | 33 (16.5) | 27 (23.9) |
| Respiratory related reintubation, n (%) | 35 (8.8) | 64 (30.7) | <.001 |
| HFNC group, n (%) | 11 (2.8) | 38 (18.3) |
| NIV group, n (%) | 24 (6.1) | 26 (12.5) |

HFNC = high-flow nasal cannula; MV = mechanical ventilation; NIV = noninvasive ventilation.

**5.- e-Table 5:** Multivariate logistic regression for reintubation according to every risk factor in the entire population, the HFNC and NIV groups separately.

| Risk factor | Entire population | HFNC | NIV |
| --- | --- | --- | --- |
|  | OR (95%CI) | OR (95%CI) | OR (95%CI) |
| Prolonged MV | 1.93 (1.22 - 3.04) | 2.67 (1.35 – 5.30) | 1.46 (.77 – 2.76) |
| APACHE II | 2.09 (1.36 - 3.22) | 2.54 (1.34 – 4.79) | 1.94 (1.04 – 3.60) |
| Not simple weaning | 1.67 (1.05 – 2.67) | 1.75 (.88 – 3.45) | 1.61 (.82 – 3.14) |
| COPD | 1.62 (.97 – 2.71) | 2.41 (1.14 – 5.07) | 1.11 (.52 – 2.37) |
| Acute heart failure | 1.03 (.47 – 2.24) | 1.81 (.56 – 5.82) | .67 (.21 – 2.12) |
| ≥2 comorbidities | 1.36 (.83 – 2.22) | 1.86 (.86 – 4.01) | .99 (.51 – 1.91) |
| BMI >30 | 1.37 (.81 – 2.30) | 2.41 (1.16 – 4.98) | .67 (.29 – 1.57) |
| Age >65 y | 1.06 (.68 – 1.65) | 1.24 (.64 – 2.41) | .94 (.50 – 1.77) |
| Airway patency | 1.74 (.47 – 6.41) | 1.60 (.12 – 20.74) | 1.77 (.38 – 8.07) |
| Secretions management | 2.44 (1.51 – 3.95) | 2.89 (1.45 – 5.76) | 1.96 (.96 – 3.98) |

APACHE = Acute Physiology and Chronic Health Evaluation; BMI = body mass index; COPD = chronic obstructive pulmonary disease; HFNC = high-flow nasal cannula; MV = mechanical ventilation; NIV = noninvasive ventilation.

**6.- e-Figure 1:** Forest plot of the multivariate logistic regression for reintubation including all risk factors.

**7.- e-Table 6:** Multivariate logistic regression for reintubation including risk factors with significant association with reintubation (prolonged MV, APACHE II on extubation day, not simple weaning, airway patency problems and secretions management).

| Risk factor | Entire population | HFNC | NIV |
| --- | --- | --- | --- |
|  | OR (95%CI) | OR (95%CI) | OR (95%CI) |
| Prolonged MV | 1.77 (1.15 - 2.75) | 2.09 (1.12 – 3.90) | 1.53 (.82 – 2.84) |
| APACHE II | 2.19 (1.43 – 3.34) | 2.68 (1.45 – 4.96) | 1.79 (.98 – 3.25) |
| Not simple weaning | 1.65 (1.04 – 2.62) | 1.74 (.90 – 3.37) | 1.62 (.83 – 3.15) |
| Airway patency problems | 1.64 (.99 – 2.72) | 2.47 (1.21 – 5.05) | 1.12 (.53 – 2.35) |
| Secretions management | 2.40 (1.50 – 3.85) | 2.58 (1.33 – 4.99) | 2.11 (1.05 – 4.24) |

APACHE = Acute Physiology and Chronic Health Evaluation; COPD = chronic obstructive pulmonary disease; HFNC = high-flow nasal cannula; MV = mechanical ventilation; NIV = noninvasive ventilation.

**8.- e-Table 7: Baseline characteristics of overweight (BMI ≥25 Kg/m^2^) and normal or underweight (BMI <25 Kg/m^2^) patients.**

|  | BMI ≥25 kg/m^2^  (n=148) | BMI <25 kg/m^2^  (n=456) | P |
| --- | --- | --- | --- |
| Age, y, median (IQR) | 67 (55 – 76) | 69 (56 – 76) | .873 |
| Female gender, n (%) | 81 (54.7) | 308 (67.7) | .004 |
| APACHE II at ICU admission, median (IQR) | 18 (14 – 22) | 16 (14 – 21) | .087 |
| APACHE II the extubation day, median (IQR) | 11 (9 – 13) | 10 (8 – 12) | .077 |
| Number of high risk factors, median (IQR) | 3 (3 – 5) | 3 (2 – 4) | <.001 |
| Four or more high-risk factors for reintubation | 69 (46.6) | 139 (30.5) | <.001 |
| Risk factors | | | |
| Prolonged MV (>7 days), n (%) | 44 (29.7) | 177 (38.9) | .044 |
| APACHE II the extubation day, mean (±SD) | 66 (44.6) | 193 (42.4) | .642 |
| Not simple weaning, n (%) | 37 (25.0) | 123 (27.0) | .627 |
| COPD, n (%) | 37 (25.0) | 79 (17.4) | .042 |
| Acute heart failure, n (%) | 18 (12.2) | 29 (6.4) | .023 |
| Charlson score ≥2, n (%) | 113 (76.4) | 309 (67.8) | .048 |
| >65 y, n (%) | 81 (54.7) | 267 (58.6) | .414 |
| Airway patency problems, n (%) | 2 (1.4) | 15 (3.3) | .266 |
| Inability to deal with secretions, n (%) | 23 (15.5) | 109 (23.9) | .032 |
| Type of respiratory support | | | |
| High flow nasal cannula, n (%) | 74 (50.0) | 216 (47.4) | .578 |
| Noninvasive ventilation, n (%) | 74 (50.0) | 240 (52.6) |
| Comorbidites | | | |
| Arterial hypertension, n (%) | 91 (61.5) | 250 (54.8) | .156 |
| Chronic heart failure, n (%) | 46 (31.1) | 151 (33.1) | .647 |
| Neurologic disease, n (%) | 45 (30.4) | 112 (24.6) | .159 |
| COPD, n (%) | 37 (25.0) | 87 (19.1) | .121 |
| Other chronic lung disease, n (%) | 53 (35.8) | 132 (29.0) | .116 |
| Diabetes mellitus, n (%) | 59 (39.9) | 120 (26.3) | .002 |
| Cancer, n (%) | 28 (18.9) | 85 (18.6) | .940 |
| Chronic vascular disease, n (%) | 20 (13.5) | 23 (5.0) | <.001 |
| Chronic renal failure, n (%) | 18 (12.2) | 61 (13.4) | .703 |
| Chronic hepatic failure, n (%) | 8 (5.4) | 52 (11.4) | .034 |
| Other chronic conditions, n (%) | 17 (11.5) | 64 (14.0) | .429 |
| Reason for mechanical ventilation initiation | | | |
| Respiratory primary failure, n (%) | 59 (39.9) | 169 (35.1) | .294 |
| ARDS, n (%) | 15 (10.1) | 38 (8.3) | .501 |
| Community adquired pneumonia, n (%) | 20 (13.5) | 65 (14.3) | .822 |
| COPD exacerbation, n (%) | 17 (11.5) | 31 (6.8) | .067 |
| Airway patency roblems, n (%) | 2 (1.6) | 11 (2.4) | .440 |
| Non respiratory primary failure, n (%) | 99 (66.9) | 310 (68.0) | .805 |
| Neurologic primary failure, n (%) | 23 (15.5) | 120 (26.3) | .007 |
| Heart primary failure, n (%) | 31 (20.9) | 59 (12.9) | .017 |
| Trauma, n (%) | 5 (3.4) | 47 (10.3) | .009 |
| Trauma brain injury, n (%) | 4 (2.7) | 24 (5.3) | .262 |
| Surgery, n (%) | 56 (37.8) | 176 (38.6) | .869 |
| Non-scheduled surgery, n (%) | 45 (30.4) | 146 (32.0) | .714 |
| Vascular surgery, n (%) | 3 (2.0) | 4 (.9) | .371 |
| Trauma surgery, n (%) | 1 (.7) | 6 (1.3) | 1.000 |
| Cardio-thoracic surgery, n (%) | 0 (0) | 5 (1.1) | .341 |
| Abdominal surgery, n (%) | 31 (21.0) | 78 (17.1) | .291 |
| Facial, neck and ENT surgery, n (%) | 0 (0) | 5 (1.1) | .341 |
| Neurosugical surgery, n (%) | 18 (12.2) | 63 (13.8) | .608 |
| Other, n (%) | 5 (3.4) | 19 (4.2) | .811 |

**9.- e-Table 8: Main outcomes (reintubation and postextubation respiratory failure) according to the stratification of the BMI (≥25 vs <25).**

|  | BMI ≥25 kg/m^2^  (n=148) | BMI <25 kg/m^2^  (n=456) | P |
| --- | --- | --- | --- |
| All cause reintubation, n (%) | 36 (24.3) | 91 (20.0) | .262  .012  .282 |
| HFNC group, n (%) | 25 (33.8) | 42 (19.4) |
| NIV group, n (%) | 11 (14.9) | 49 (20.5) |
| Respiratory related reintubation, n (%) | 35 (23.6) | 64 (14.0) | <.001 |
| HFNC group, n (%) | 11 (7.4) | 38 (8.3) |
| NIV group, n (%) | 24 (16.2) | 26 (5.7) |

**10.- e-Table 9:** Multivariate logistic regression for reintubation in overweight patients adjusted for covariates according to therapy.

| Risk factor | HFNC | | NIV | |
| --- | --- | --- | --- | --- |
|  | OR (95%CI) | P | OR (95%CI) | P |
| BMI ≥25 | 2.47 (1.18 – 5.15) | .016 | .61 (.27 – 1.39) | .242 |
| ≥4 risk factors | 2.46 (1.01 – 5.96) | .047 | .81 (.34 – 1.90) | .637 |
| APACHE II >12 | 1.07 (.96 – 1.19) | .178 | 1.15 (1.02 – 1.29) | .020 |
| Secretions management | 2.46 (1.15 – 5.28) | .020 | 2.66 (1.26 – 5.64) | .010 |
| ≥2 comorbidities | 1.55 (.63 – 3.79) | .329 | .72 (.33 – 1.58) | .423 |
| Acute heart failure | 2.27 (.40 – 12.83) | .35 | .90 (.20 – 4.06) | .901 |
| COPD | 2.05 (.78 – 5.36) | .142 | 1.56 (.58 – 4.15) | .373 |
| Prolonged MV | 2.56 (1.20 – 5.48) | .015 | 1.66 (.83 – 3.31) | .148 |
| Female gender | 1.24 (.61 – 2.54) | .542 | .72 (.37 – 1.38) | .327 |
| Chronic hepatic disease | 1.56 (.58 – 4.18) | .368 | 1.99 (.70 – 5.59) | .191 |
| Vascular disease | .95 (.30 – 3.01) | .940 | 3.02 (.98 – 9.26) | .053 |
| Diabetes Mellitus | 1.21 (.57 – 2.52) | .612 | 1.42 (.68 – 2.94) | .340 |
| COPD exacerbation | .54 (.12 – 2.46) | .434 | .48 (.12 – 1.91) | .301 |
| Trauma | 3.55 (1.09 – 11.6) | .035 | 1.05 (.36 – 3.08) | .919 |
| Hemodynamic failure | .52 (.14 – 1.93) | .331 | .53 (.17 – 1.65) | .276 |
| Neurologic failure | 1.95 (.86 – 4.41) | .104 | 1.10 (.51 – 2.37) | .795 |

**11.- e-Table 9:** Effect modification on reintubation rate according to the number of risk factors.

|  | NIV  (n=314) | HFNC (n=290) | NIV vs HFNC | |
| --- | --- | --- | --- | --- |
| Nº risk factors | Reintubated / total nº patients | Reintubated / total nº patients | RD (95%CI)^1^ | RR (95%CI)^2^ |
| 1 | 4/39 | 3/36 | -1.9 (-16.3 – 12.9) | .81 (.2 – 3.4) |
| 2 | 10/78 | 9/80 | -1.6 (-12.1 – 8.9) | .88 (.4 – 2.1) |
| 3 | 19/83 | 12/80 | -7.9 (-19.8 – 4.3) | .65 (.3 – 1.3) |
| 4 | 14/64 | 16/47 | 12.2 (-4.4 – 28.7) | 1.56 (.8 – 2.8) |
| 5 | 6/34 | 13/31 | 24.3 (2.1 – 43.9) | 2.38 (1.1 – 5.6) |
| 6 | 5/12 | 10/13 | 35.3 (-2.6 – 52.1) | 1.84 (.9 – 3.8) |
| 7 | 2/4 | 3/3 | 50 (-16.2 - 85) | 1.86 (.8 – 4.1) |

NIV=noninvasive ventilation; HFNC=high-flow nasal cannula; RD=risk difference; RR=relative risk; RRR=ratio of relative risk; RERI_RR_=relative excess risk due to interaction

^1^Global RD (risk difference or interaction contrast)

^2^R+ (risk plus) = 5·4% (1% to 9·7%), p=0·016

RRR = 1·33 (95%CI 1·13 to 1·56), p<0·001

RERI_RR_ = 0·12 (3E^(-4)^ to 0·24), p=0·049

**e-Figure 2:** Effect modification on an additive scale: 5.4-fold increase in risk difference for reintubation rate for each additional risk factor.

**
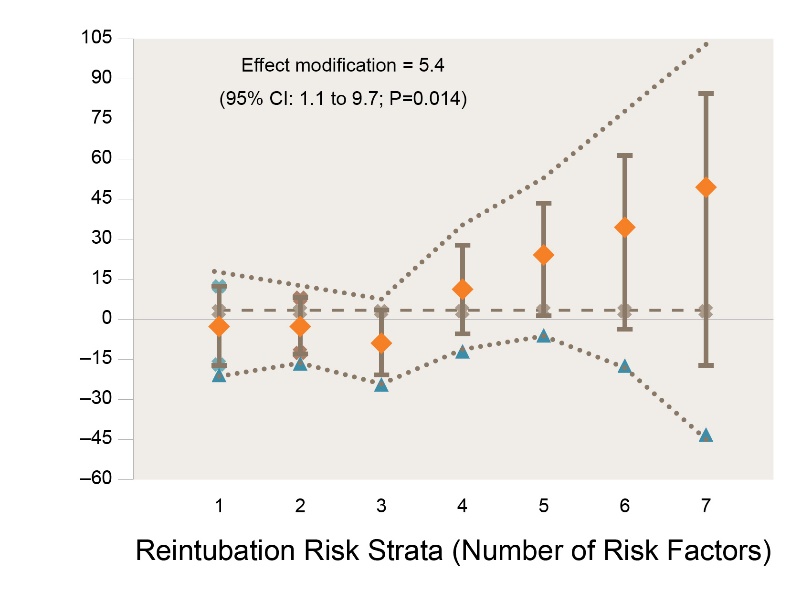
**


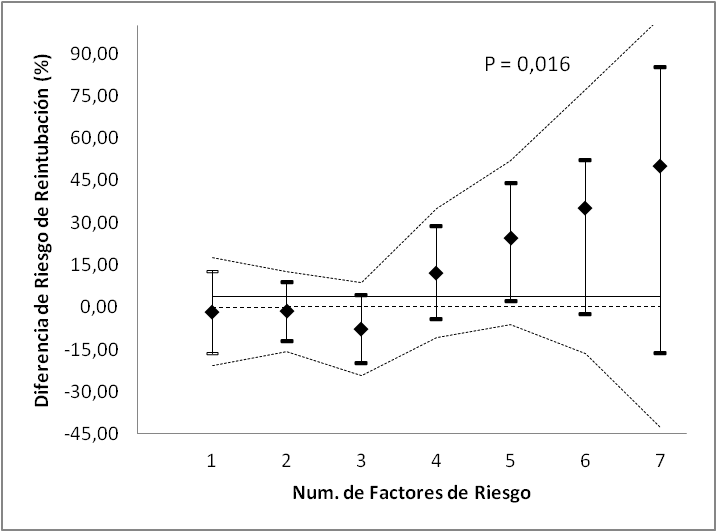
Horizontal line: Risk difference (RD) marginal to risk strata (3.65% in the original study).

Diamonds: RD with successive increments of one risk factor.

Vertical lines: point-wise RD 95% CI. Dotted lines: Simultaneous RD 95%CI limits.

**e-Figure 3:** Effect modification on a multiplicative scale: The relative risk (RR) ratio increases 1.33-fold for each additional risk factor.


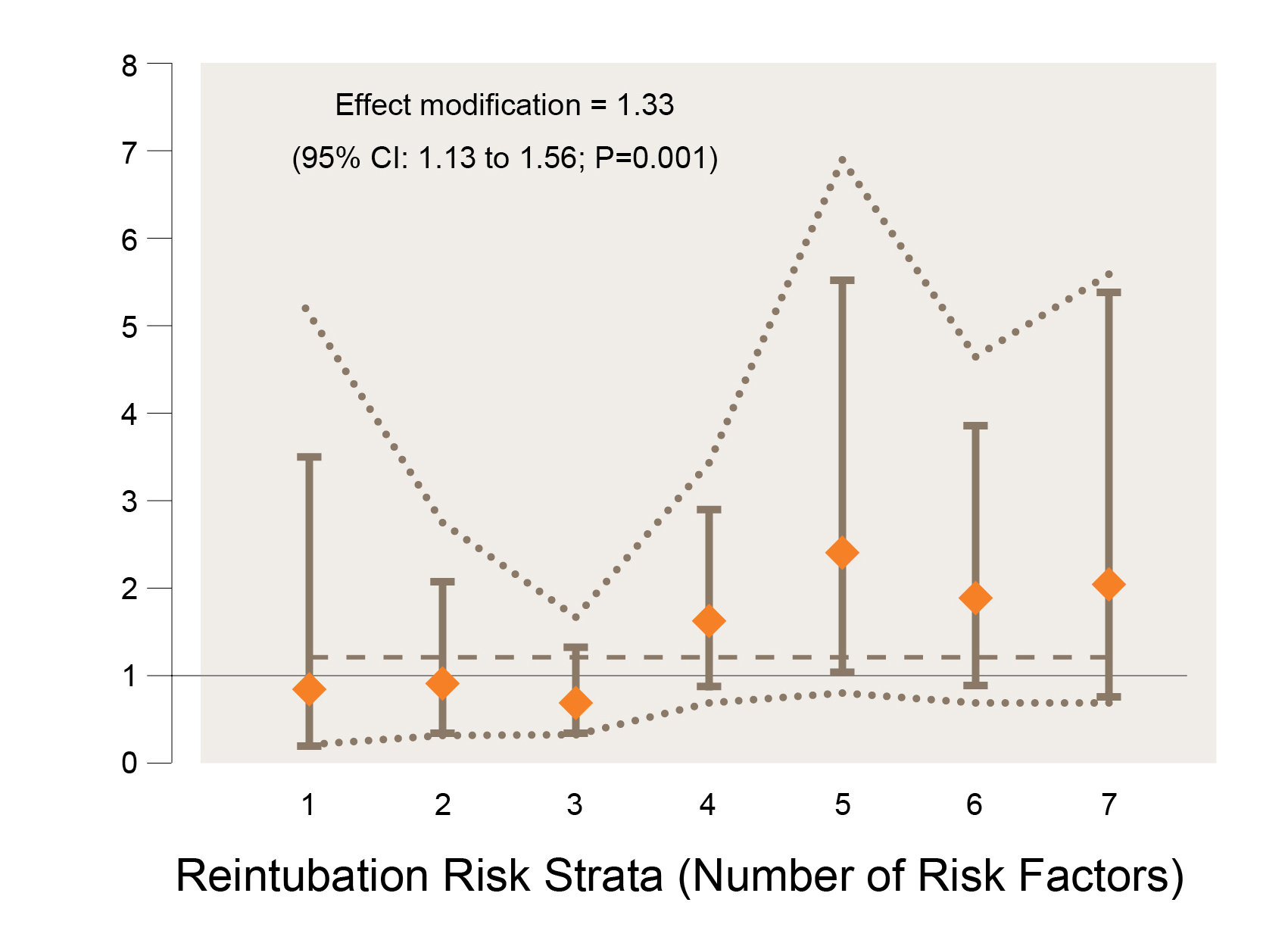


Horizontal line: RR ratio marginal to the risk stratum (1.19 in the original study).

Diamonds: Ratio of RR in each stratum.

Vertical lines: Pointwise ratio of RR 95%CI.

Dotted lines: Simultaneous ratio of RR 95%CI limits.
